# Are Social and Communication Difficulties a Risk Factor for the Development of Social Anxiety?

**DOI:** 10.1016/j.jaac.2017.01.007

**Published:** 2017-04

**Authors:** Hannah Pickard, Fruhling Rijsdijk, Francesca Happé, William Mandy

**Affiliations:** a Medical Research Council (MRC) Social, Genetic, and Developmental Psychiatry (SGDP) Centre, King’s College London, UK; b University College London, UK

**Keywords:** ALSPAC, social anxiety, social and communication difficulties, autism spectrum disorders, longitudinal

## Abstract

**Objective:**

Social anxiety (SA) is a common condition associated with social and communication (SC) difficulties in typically developing young people, as well as those with autism spectrum disorder (ASD). Whether SC difficulties place children at risk for developing SA is unclear. Using a longitudinal design, the present study aimed to disentangle the relationship between SA symptoms and SC difficulties using a population-based sample of 9,491 children from the Avon Longitudinal Study of Parents and Children (ALSPAC).

**Method:**

Parent-reported data on SC difficulties and SA symptoms were collected at ages 7, 10, and 13 years. A cross-lagged panel model was used to investigate the longitudinal stability and directional relationship between latent SC difficulties and SA constructs over time.

**Results:**

More SC difficulties were associated with greater SA symptoms at all ages. Earlier SC difficulties predicted a small but significant amount of variance in later SA symptoms. The reverse relationship from SA to SC difficulties was not observed. The relationship from SC difficulties to SA was strongest from age 7 to 10 years. No sex differences were observed.

**Conclusion:**

The evidence suggests that SC difficulties may be an important risk factor for the development of SA. These findings suggest the potential usefulness of incorporating social skills training alongside effective interventions to prevent or alleviate symptoms of SA in childhood.

Social anxiety (SA) disorder is characterized by an intense fear of social situations, which is often accompanied by the fear of being scrutinized by others.^1^ SA is often experienced during several social situations, including interacting with others, eating in public, or giving speeches. Anxiety-related fears are commonly driven by negative self-perceptions and a fear of being ridiculed by others, which can lead to increased social withdrawal and avoidance.^2^ SA is the third most common psychiatric disorder, with epidemiological research showing prevalence rates between 3% to 4% in childhood and 9% in adolescence.^3^, ^4^ The onset of SA is usually between late childhood and adolescence^5^; however, SA disorder can manifest in children at 7 or 8 years of age.^6^

SA is a dimensional trait that is continuously distributed throughout the general population. Subthreshold symptoms of SA are associated with adverse outcomes and an increased risk of developing SA disorder and additional comorbid disorders.^7^ Given the burden that subthreshold SA can have on an individual’s well-being, it is important that research investigates potential risk factors underpinning dimensionally measured SA traits in the general population.

### SA and Social Communication Difficulties in Childhood

Etiological models of SA in childhood have implicated the role of several development risk factors, including behavioral inhibition, parent–child interactions, and peer relationships.^8^ In addition, social and communication (SC) difficulties, including problems in social behaviors, social cognition, and reciprocal social communication, are common among children with SA and have also been proposed as a risk factor. SC ability is a continuously distributed trait that extends throughout the general population,^9^ with those who experience severe difficulties often receiving a diagnosis of autism spectrum disorder (ASD), a neurodevelopmental condition characterized by SC difficulties and restricted interests and repetitive behaviors. SA co-occurs highly in children with ASD (4.5−9.5 years) and high subthreshold ASD traits (10−15 years),^10^, ^11^ suggesting that those with greater SC difficulties may have a heightened risk of developing SA disorder. However, the developmental relationship between SC difficulties and SA is unclear. The present study aims to address whether an individual’s position on the continuum of SC traits influences their risk of later developing SA.

Cross-sectional research has supported the association between SC difficulties and SA. For example, typically developing children with SA disorder exhibited lower self and peer ratings of social competence during both laboratory and school-based social interaction tasks, compared to peers without anxiety.^4^, ^12^ Furthermore, using parent-report questionnaires, research has found that SC difficulties are higher among children with SA disorder compared to those with other anxiety disorders,^13^ suggesting a specific relationship between SC difficulties and SA. Although SC difficulties may not be universal in SA,^14^ the evidence suggests that for a significant subgroup in the general population, SC difficulties may underlie the development of SA.^15^

Intervention studies in children have informed our understanding of the relationship between SC difficulties and SA. Research has shown that children (age 8−12 years) with SA disorder who completed a Social Effectiveness Therapy (SET) program to enhance social skills and peer relationships showed increases in social skills and decreases in SA at posttreatment and after 6 months, compared to the control participants in a nonspecific intervention.^16^ It is evident that a relationship between SA and SC difficulties exists and that social skills training effectively reduces SA; however, we do not fully understand whether SC difficulties contribute to the development of SA. This research is important for identifying early warning signs on the developmental trajectory of SA.

In the ASD literature, cross-sectional studies have shown that social skill deficits and greater physiological arousal contributed toward elevated SA symptoms in adolescents with ASD.^17^ Contradictory research in children with ASD revealed that higher levels of SA predicted lower responsible and assertive social skills.^18^ Inconclusive findings regarding the directional relationship between SA and SC difficulties have led researchers to postulate a bidirectional relationship in ASD. It is suggested that SC difficulties may hinder social experiences, contributing to increased SA and social withdrawal, which subsequently impedes an individual’s SC ability.^19^ However, this relationship remains to be explored.

Research using population-based samples has supported the relationship between SA symptoms and SC difficulties. Population-based research allows the use of large samples to examine associations across the trait distribution. These findings can inform research in clinical populations. Using a population-based twin sample of children with ASD and their affected and unaffected cotwins, Hallett *et al.*^11^ found that increased SC difficulties and higher IQ were associated with greater parent-reported SA, supporting clinical findings. To date, no longitudinal work using a population-based sample has specifically assessed whether SC difficulties are a risk factor for SA, or whether SA reduces an individual’s SC ability.

We aim to examine the directional relationship between parent-reported SA symptoms and SC difficulties in a population-based sample of children at ages 7, 10, and 13 years. Sex differences will also be explored. Furthermore, the relationship between SA and SC difficulties will be examined while controlling for generalized anxiety, to test whether SC difficulties are related to SA-specific symptoms, compared to generalised anxiety. Based on previous research, we predict a directional and specific relationship between SC difficulties and SA, with early SC difficulties contributing to later SA symptoms.

## Method

### Sample

All participants were from the Avon Longitudinal Study of Parents and Children (ALSPAC) cohort, a population-based sample of children born in Bristol between 1991 and 1992. A total of 14,541 children were recruited into the original cohort, with 14,062 live births and 13,988 alive at 12 months.^20^ (The study website contains details of all of the data available through a fully searchable data dictionary at http://www.bris.ac.uk/alspac/researchers/data-access/data-dictionary/.) Ethical approval for this study was obtained from the ALSPAC Law and Ethics Committee and local research ethics committees. A total of 9,597 children had available data to test the study hypotheses at ages 7 (n = 8,148), 10 (n = 7,723), and 13 (n = 7,008) years. Following ALSPACs exclusion criteria for prorated scores, only children with 50% or more complete data on all measures of interest at all ages were included in the present study. Based on these exclusion criteria, 248 children (3%), 204 children (2.6%), and 226 children (3.2%) were excluded at ages 7, 10, and 13 years, respectively. Merging the three samples at ages 7 (n = 7,900), 10 (n = 7,519), and 13 (n = 6,782) years, the final sample included 9,491 children (4,654 female) with data at one, two, or three time points. This final sample was used in all further analyses (Table 1). Compared to the original ALSPAC cohort not included in the current analyses, young people in our final sample were more likely to have a mother who was a homeowner (odds ratio [OR] = 2.94, 95% CI = 2.72, 3.19) and had completed higher education (OR = 2.33, 95% CI = 2.13, 2.55).

### Measures

#### Socioeconomic Status

Socioeconomic status (SES) was captured using parental maternal education. Previous research in ALSPAC has reported that maternal education is a valid indicator of SES.^21^ At 32 weeks of gestation, mothers reported their current highest level of educational achievement from six possible responses: “none,” “CSE” (basic UK qualification), “vocational,” “O-level” (a prerequisite to further education), “A-levels,” and “degree or above.” Higher scores are indicative of better maternal education and thus higher SES.

#### Wechsler Intelligence Scale for Children−Third Edition

The Wechsler Intelligence Scale for Children−Third Edition (WISC-III)^22^ is a measure of child IQ. In the present study, the abbreviated version of the WISC, including random items from 10 subtests, was administered during the clinical data collection wave at age 8 years. A total of 6,726 children (70.9%) from the final sample had a complete IQ measure at age 8 years.

#### Social and Communication Disorders Checklist

The Social and Communication Disorders Checklist (SCDC)^23^, ^24^ is a parent-reported questionnaire that measures social and communication (SC) difficulties related to ASD. The questionnaire consists of 12 items with a response scale ranging from 0 to 2 (“not true,” “quite or sometimes true,” “very often true”), which was designed to capture a child’s social behavior and functioning over the previous 6 months. A total score ranges from 0 to 24, with higher scores indicating greater SC difficulties. The SCDC shows high internal consistency (0.93), as well as good specificity (0.91) and sensitivity (0.88) when discriminating between individuals with and without ASD.^23^ Furthermore, research conducted in the ALSPAC cohort supports both the construct validity and reliability of the SCDC at measuring SC traits in the general population.^24^ In the ALSPAC sample, research has shown that the SCDC measures SC trait variability in the general population that overlaps with ASD in terms of genetic effects,^25^ supporting the SCDC’s validity as a measure of ASD-specific SC difficulties. The SCDC had excellent internal reliability (α = 0.81−0.89).

#### Development and Wellbeing Assessment

The Development and Wellbeing Assessment (DAWBA)^26^ questionnaire was administered as a parent-report questionnaire to capture child and adolescent psychopathology that corresponds with the *International Classification of Diseases–10*^*th*^ *Revision* (*ICD-10*) and *Diagnostic and Statistical Manual of Mental Disorders—4th Edition (DSM-IV)* criteria. The DAWBA has been tested and validated in large population samples.^26^ In the present research, SA symptoms were measured using the social fears (SF) subscale, and generalized anxiety was measured using the general anxiety (GA) subscale. The DAWBA-SF has six items in which parents report whether their child had experienced any specific SA symptoms over the last month: “no,” “a little,” “a lot,” and “hasn’t done this in last month.” Any parent responses of “hasn’t done this in last month” were excluded, as this response is not present in the original online DAWBA and is ambiguous in its answer to the six SF items. An SF total score (range 0−12) can be created by summing the responses over the six SA items, which was used in the present study. Higher scores on the DAWBA-SF indicate more severe SA symptoms. The DAWBA-SF showed good internal reliability (α = 0.79−0.81). The DAWBA-GA subscale consists of seven items in which parents report the frequency of their child worrying over the past 6 months: “no,” “sometimes,” and “often.” A GA total score (range 0−14) is computed by summing responses on all items, with higher scores indicating more generalized anxiety symptoms. The DAWBA-GA showed acceptable internal reliability (α = 0.53−0.72).

### Data Analyses

Analyses were conducted in R, using the Lavaan package for structural equation modeling (SEM).^27^ The present study used a three-wave (time), two-level cross-lagged panel model to estimate relationships between SC difficulties and SA symptoms. The cross-lagged panel model incorporates the inherent time nature of longitudinal data and is frequently used to assess causal relationships in nonexperimental studies using panel data.^28^, ^29^

#### Confirmatory Factor Analyses

Three confirmatory factor analyses were conducted to assess the construct validity of the DAWBA-SF and SCDC at all ages. A two-factor structure was specified with a single factor for each scale: SC difficulties (SCDC) with 12 indicators, and SA (DAWBA-SF) with 6 indicators. Measures recommended for large datasets were used.^30^ Absolute fit measures included the standardized root mean square residual (SRMR) and root mean square error of approximation (RMSEA). For the SRMR, a value less than 0.08 indicates a good model fit, and for RMSEA, a value below 0.08 indicates an acceptable model fit, with values less than 0.05 indicating good model fit.^31^, ^32^ The comparative fit index (CFI) was also used, with values above 0.90 and 0.95 indicating acceptable to good model fit, respectively.^32^

#### Cross-Lagged Panel Model

**Figure 1:**
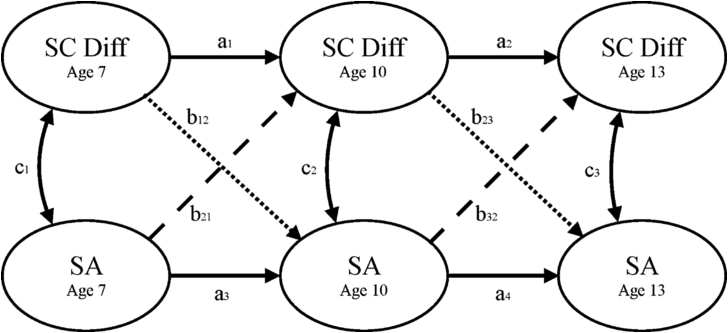
Cross-lagged panel model of social and communication difficulties (SC Diff) and social anxiety (SA) latent factors. Note: A = autoregressive paths, b = cross-lagged paths; c = covariance paths.

The predicted relationships between SC difficulties and SA symptoms at ages 7, 10, and 13 years are depicted in Figure 1. The simultaneously solved paths are reported as partial regression coefficients: autoregressive paths estimate the stability of one trait over time; covariance paths estimate the correlation between two traits at each time point; cross-lagged paths estimate the predictive relationship of one variable on another at a later time point, independent of the stability and covariance paths.

The relationships were tested between latent factors to capture more robust constructs free of measurement error. Each latent factor was specified within the model, with the 6 items from the DAWBA-SF loading on to the SA construct and the 12 items from the SCDC loading on to the SC difficulties construct (excluded from Figure 1 for simplicity). In the full model, latent factors were free to covary within time points. Latent factor item residuals were specified to covary between time points.

Model fit was tested with the Satorra–Bentler Scaled χ^2^ statistic,^33^ to compare χ^2^ when data are nonnormal. To test the study’s first hypothesis, model fit across 8 nested models were examined to assess the following: longitudinal stability of each latent variable; the relationship between the latent variables within time; and the stability of cross-lagged paths and difference in the cross-lagged paths at each time point (7→10, 10→13). Model fit was determined by the difference in fit statistics of the full model and a nested model in which equality constraints are applied to path estimates (e.g., a_1_ = a_2_ or a_3_ = a_4_ to assess stability of the autoregressive paths). To assess sex differences, likelihood ratio testing was conducted between a full model in which all paths were freely estimated across sex and one in which either all cross-lagged paths (b_12_, b_21_, b_23_, b_32_) or autoregressive paths (a_1_, a_2_, a_3_, a_4_) were equated across sex. A Bonferroni correction was applied to assess the significance of all path coefficients (*p* < .003). χ^2^ for model-fit differences were considered to be statistically significant at *p* < .006.

#### Specificity

To explore the specificity of the relationship between SC difficulties and SA, scores on the DAWBA-GA subscale (where available) were regressed out of the SA latent variable traits at ages 7, 10, and 13 years to create a more specific SA-related latent construct.

## Results

All questionnaire data were cleaned using ALSPAC guidelines for data preparation. Tests of selective attrition for SC difficulties and SA symptoms were conducted, and acceptable results were observed (see Supplements 1 and 2, available online). Mean scores on the SCDC and DAWBA-SF are reported in Table 2. The full distribution of scores on the SCDC and DAWBA-SF scales are available online (see Table S1, available online).

### Confirmatory Factor Analysis

Three two-factor models were specified to test the construct validity of the SCDC scale and DAWBA-SF subscale at ages 7, 10, and 13 years. Two out of three of the fit indices for the two-factor models at 7 years (RMSEA = 0.067 [0.065, 0.068], SRMR = 0.06, CFI = 0.83), 10 years (RMSEA = 0.067 [0.066, 0.069], SRMR = 0.06, CFI = 0.84), and 13 years (RMSEA = 0.073 [0.072, 0.074], SRMR = 0.06, CFI = 0.82) were indicative of a good/acceptable model fit. The results imply that the SCDC and DAWBA-SF are two distinct and separate constructs measuring SC difficulties and SA symptoms.

### Latent Variable Correlations

A saturated model with no cross-lagged or stability paths was fitted to examine the correlations among all latent factors. Significant associations among all latent factors were observed (Table 3).

### Cross-Lagged Panel Model Path Estimates

In the cross-lagged panel model, effects of IQ and SES were regressed out of the SA latent variable at ages 7 years (IQ: β = −0.18, SES: β = −0.02) 10 years (IQ: β = −0.11, SES: β = 0.01), and 13 years (IQ: β = −0.04, SES: β = 0.00). For the SC difficulties latent variable, the effects were at ages 7 years (IQ: β = −0.17, SES: β = 0.00), 10 years (IQ: β = −0.11, SES: β = −0.00), and 13 years (IQ: β = −0.04, SES: β = −0.00).

### Covariance Paths

**Figure 2:**
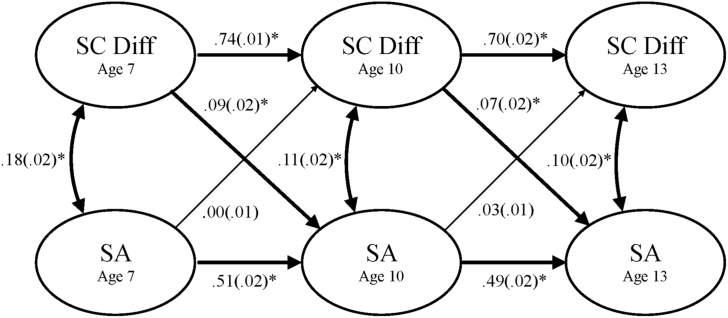
Cross-lagged panel model showing the relationship between social and communication difficulties (SC Diff) and social anxiety (SA) latent factors at 7, 10, and 13 years of age. Note: Standardized β coefficients with standard errors. All analyses controlled for IQ and socioeconomic status (SES). Significant paths are shown in bold. ∗*p* < .001.

The covariance path estimates between SC difficulties and SA were significant at all ages (Figure 2). The covariance path weights steadily decreased over time; however, no significant decrease in model fit was observed when the covariance paths at ages 7 and 10 years (Δχ^2^[df] = 4.92[1], *p =* .03) and ages 10 and 13 years (Δχ^2^[df] = 0.12[1], *p =* .73) were constrained to be equal (see Table S2, available online).

### Stability Paths

The autoregressive paths for SC difficulties (a_1_ and a_2_) and SA (a_3_ and a_4_) were significantly stable over time. However, the longitudinal stability of both SC difficulties and SA significantly decreased over time: SC difficulties (Δχ^2^[df] = 53.87[1], *p =* 2.15^e-13^) and SA symptoms (Δχ^2^[df] = 12.16[1], *p =* 4.89^e-04^).

### Cross-Lagged Paths

The cross-lagged paths from SC difficulties to SA (b_12_ and b_23_) were both significant, but not significantly different in size (Δχ^2^[df] = 4.52[1], *p =* .03). The reverse cross-lagged paths from SA to SC difficulties (b_21_ and b_32_) were not significant. Subsequent analyses explored the difference in cross-lagged path weights at ages 7→10 years and 10→13 years. A significant difference in the cross-lagged paths from age 7→10 years was observed (Δχ^2^[df] = 13.04[1], *p =* 3.06^e-04^), with the path from SC difficulties to SA having a significantly greater contribution compared to the reverse cross-lagged path. No significant difference was seen for the cross-lagged paths from age 10→13 years (Δχ^2^[df] = 1.06[1], *p =* .30).

### Sex Differences

No significant decrease in model fit was observed for a nested model constraining all cross-lagged paths to be equal across male and female participants (Δχ^2^[df] = 1.59[4], *p =*.81), compared to a full model, indicating no sex differences in the predictive relationship between SA and SC difficulties constructs at all ages. Analyses investigating sex differences in the longitudinal stability showed a significant difference in the autoregressive pathways for SC difficulties (Δχ^2^[df] = 22.68[2], *p =* 1.19^e-05^), with females showing less stability in SC difficulties compared to males. No sex differences were observed for the SA autoregressive paths (Δχ^2^[df] = 4.61[2], *p =* .10).

### Specificity Analyses

Specificity analyses tested the relationship between SC difficulties and SA, while controlling for generalized anxiety (see Table S3, available online). The analyses revealed a pattern of results identical to that of the full cross-lagged panel model, showing both significant autoregressive paths and significant cross-lagged paths from SC difficulties to SA at ages 7→10 and 10→13 years. The reverse cross-lagged paths from SA to SC difficulties were not significant.

## Discussion

We used a longitudinal design to investigate the relationship between SC difficulties and SA symptoms in a population-based cohort of children at ages 7, 10, and 13 years. We predicted that SC difficulties would contribute specifically to the development of SA symptoms in later childhood. We found that, first, more parent-reported SC difficulties were associated with heightened SA symptoms across all ages. Second, the data supported the construct validity of the SCDC and DAWBA-SF, suggesting that SA and SC difficulties are distinct domains across childhood. Third, extending previous research and supporting our predictions, we found a directional and asymmetrical relationship between SC difficulties and SA symptoms; earlier SC difficulties contributed toward the development of later SA symptoms, but not vice versa. In terms of this directional relationship, sex differences were not observed. Finally, SC difficulties predicted later SA symptoms while controlling for generalized anxiety, emphasizing that SC difficulties are a specific risk factor for SA. The interpretation of these results, clinical implications, limitations, and conclusions are discussed below.

In typically developing children, associations between clinical SA symptoms and poorer social skills have been reported.^4^, ^12^ Our results both support and extend previous findings by illustrating the stability of these relationships throughout childhood. In accordance with research reporting more SC difficulties and greater SA symptoms in individuals with ASD,^11^, ^13^ we found similar associations in a population-based sample of children. The magnitude of these associations, although only modest compared to results in clinical samples,^11^ mimic the findings from previous traitwise research examining parent-reported SC difficulties and SA symptoms.^34^ Our results may be indicative of the low levels of SA and SC difficulty scores in the present sample.

Previous intervention studies have supported the efficacy of social skills therapies for improving SC ability and having downstream benefits on SA.^16^ Building on this work, our study demonstrates that these SC difficulties not only co-occur with SA, but also appear to play a role in the development of SA across childhood. In addition, our novel longitudinal findings in a population-based sample suggest that SC difficulties are a risk factor for the development of SA across the trait distribution. These findings emphasize a potential marker for the development of SA that could be targeted with early prevention approaches.

Furthermore, our results are consistent with etiological theories proposing that SC difficulties may provoke negative reactions from others, which, through repeated experience, may result in increased SA.^15^ This is one possible mechanism through which SC difficulties may predispose to greater SA symptoms in childhood; however, there may be several alternative mechanisms, for example peer victimization, bullying, or social insight,^35^ that may contribute to the development of SA in those who exhibit severe SC difficulties. For example, in adolescents with ASD, self-reported peer victimization and bullying are associated with increased internalizing problems.^36^ It is possible that SC difficulties predispose to these additional risk factors or that they develop independent of social ability. Further research exploring the mediating mechanisms on the developmental pathway from SC difficulties to SA in childhood is warranted.

Interestingly, SC difficulties in earlier childhood made a greater contribution to SA symptoms, compared to the alternative cross-lagged path from age 7 to 10 years, suggesting that earlier SC problems heighten a child’s risk of developing SA. In support of this, our model results show that the strongest association between SC difficulties and SA was present at age 7 years. The greater impact of SC difficulties, particularly in earlier childhood, may explain the high prevalence rates of SA disorder in children with ASD,^10^ where SC difficulties arise earlier in development. Given these findings and that SA is common among children as young as 7 and 8 years of age,^4^ it is important for further research to investigate whether SC difficulties predict SA in younger children.

In late childhood, no difference in the strength of the cross-lagged paths was observed. The decrease in the difference of predictive magnitude at ages 10 to 13 years may reflect the influence of additional risk factors. These risk factors may be exacerbated by SC difficulties (e.g., bullying or peer neglect) or independent of these abilities (e.g., traumatic events), but contribute toward the development of SA. Alternatively, age-specific socioemotional and physical changes in late childhood/adolescence, including puberty and increased social pressures, may contribute to SA development. These developmental changes bring with it increased feelings of anxiety, stress, and social pressures, with more opportunities to misjudge social situations and experience social failure, a process that could be exacerbated by an individual’s social disability and insight into their ability.^37^ In fact, high-functioning adolescents with ASD who may have greater insight into their social ability exhibit greater SA symptoms.^38^ Coupled with SC difficulties, greater social insight may play an important role in the onset of SA in late childhood. Alternatively, a lack of social insight may be a protective factor against the development of SA, as these individuals may simply not care about what others think about them. Research exploring this process requires further investigation.

Our results revealed no sex differences in the pattern of directional relationships, despite epidemiological research reporting high prevalence rates of SA in females.^39^ Interestingly, although girls who experience severe SC difficulties come to clinical attention less often than boys,^40^ our results suggest that girls with SC difficulties are just as likely as boys to suffer the negative consequences of these impairments, in terms of SA. Further research examining sex differences in the risk factors for SA is warranted, as important sex differences may emerge.

The specificity analysis revealed that SC difficulties are a specific risk factor for childhood SA symptoms, over and above generalized anxiety. Supporting evidence shows that children with SA disorder exhibit greater parent-reported SC difficulties, compared to children with other anxiety and mood disorders.^13^ The combined evidence highlights the specificity of the relationship between SC difficulties and SA symptoms, compared to other forms of anxiety.

The present study was strengthened by its large sample size and the use of consistent measures throughout childhood. However, limitations should be considered. First, all analyses relied on parent-report measures, and future research would benefit from a multi-informant approach. Second, the DAWBA-SF scale fails to capture several physiological, cognitive, and behavioral symptoms and relies on six items, suggesting a lack of richness compared to other clinical measures. In addition, the DAWBA has a tendency to overdiagnose emotional disorders,^41^ so it is possible that SA levels are elevated in this sample. Novel and comprehensive measures of IQ, SA, and SC difficulties would be beneficial in future research. Finally, the cross-lagged model design is subject to many limitations,^42^ and the modest correlations between SC difficulties and SA suggest that additional factors may influence this relationship over time, an interesting avenue for future research.

In conclusion, our findings demonstrate that SC difficulties in early childhood contribute toward the development of later SA symptoms, but not vice versa. In light of these novel findings, clinical implications are considered. Previous research using social skill interventions based on cognitive-behavioral therapy approaches have been effective at both increasing social skills and decreasing symptoms of anxiety in adolescents with ASD.^43^ Building on this work, our results support the use of social skill programs alongside gold standard interventions in childhood, which offers the opportunity to develop SC skills while simultaneously improving symptoms of SA. Furthermore, given the focus of social cognition in our measure of SC difficulties, our findings imply that improving social cognition may be a key target for SA interventions. Using dual treatment programs that target social skills and potentially social cognition in childhood could be an effective route to alleviate symptoms of SA.

## Figures and Tables

**Table S1:** Number of Children Who Fall Within the Distribution Bands of Scores on the Social Communication Disorders Checklist (SCDC) and Development and Wellbeing Assessment Social Fears Subscale (DAWBA-SF) at 7, 10, and 13 Years

Questionnaires	Age 7 y (n = 7,900)	Age 10 y (n = 7,519)	Age 13 y (n = 6,782)
	n (%)	n (%)	n (%)
SCDC (Range: 0−24)^a^			
0−3	5,608 (71.0)	5,757 (76.6)	4,993 (73.6)
4−7	1,534 (19.4)	1,125 (15.0)	1,171 (17.3)
8−12	525 (6.6)	419 (5.6)	441 (6.5)
13−18	158 (2.0)	152 (2.0)	124 (1.8)
18−24	75 (0.9)	66 (0.9)	53 (0.8)
DAWBA-SF (Range: 0−12)^a^			
0−3	7,282 (92.2)	6,854 (91.2)	5,945 (87.7)
4−7	558 (7.1)	590 (7.8)	742 (10.9)
8−12	60 (0.8)	75 (1.0)	95 (1.4)

Note:

a Full range of scores at all ages.

**Table S2:** Cross-Lagged Panel Model Path Estimates Between Social and Communication (SC) Difficulties and Social Anxiety (SA) Latent Constructs and Fit Statistics for Constrained Model Paths Estimates

Model Paths	B (SE)	95% CI	β (SE)	*p*	Model Comparisons	χ^2^ (*df*)	AIC	Δχ^2^ (df)	*p*
					Full Model	26604.13 (1408)	376308.86	—	—
Covariance Paths					Covariance Paths				
c_1_	—	—	0.18 (0.02)	**0.00^e+00^**	c_1_ = c_2_	26613.72 (1409)	1017001.48	4.92 (1)	.03
c_2_	—	—	0.11 (0.02)	**7.27^e-07^**	c_2_ = c_3_	26604.36 (1409)	1016992.12	0.12 (1)	.73
c_3_	—	—	0.10 (0.02)	**4.64^e-07^**					
Autoregressive Paths					Autoregressive Paths				
a_1_	1.11 (0.04)	[1.03, 1.20]	0.74 (0.01)	**<2^e-16^**	a_1_ = a_2_	26793.46 (1409)	376496.20	53.87 (1)	**2.15^e-13^**
a_2_	0.67 (0.03)	[0.61, 0.73]	0.70 (0.02)	**<2^e-16^**	a_3_ = a_4_	26629.72 (1409)	376332.45	12.16 (1)	**4.89^e-04^**
a_3_	0.61 (0.03)	[0.56, 0.67]	0.51 (0.02)	**<2^e-16^**					
a_4_	0.48 (0.02)	[0.44, 0.53]	0.49 (0.02)	**<2^e-16^**					
Cross-Lagged Paths					Cross-Lagged Paths				
b_12_	0.11 (0.02)	[0.07, 0.15]	0.09 (0.02)	**1.00^e-07^**	b_12_ = b_23_	26612.25 (1409)	376314.98	4.52 (1)	.03
b_21_	0.00 (0.02)	[−0.03, 0.04]	0.00 (0.01)	8.29^e-01^	b_21_ = b_32_	26605.40 (1409)	376308.13	0.86 (1)	.35
b_23_	0.05 (0.01)	[0.02, 0.08]	0.07 (0.02)	**2.31^e-04^**	b_12_ = b_21_	26624.15 (1409)	1017011.91	13.04 (1)	**3.06^e-04^**
b_32_	0.03 (0.02)	[−0.00, 0.06]	0.03 (0.01)	7.40^e-02^	b_23_ = b_32_	26605.66 (1409)	1016993.42	1.06 (1)	.30

Note: Standardized β coefficients and unstandardized B coefficients are reported with standard errors (SE). 95% CIs are reported for standardized B coefficients. Scaled χ^2^ difference test statistic (Δχ^2^) using the Satorra–Bentler method. Maximum likelihood χ^2^ statistics are used. Exact path *p* values are reported. Bold *p* values are significant. All pathways are denoted by subscripts (see Figure 1). AIC = Akaike Information Criterion.

**Table S3:** Specificity Cross-Lagged Panel Model Path Estimates Between Social and Communication Difficulties and Social Anxiety Latent Constructs With Generalized Anxiety Scores Regressed Out

Model Paths	B (SE)	95% CI	β (SE)	*p*
Covariance Paths				
c_1_	—	—	0.13 (0.02)	**1.39^e-13^**
c_2_	—	—	0.10 (0.02)	**9.81^e-06^**
c_3_	—	—	0.09 (0.02)	**1.26^e-05^**
Autoregressive Paths				
a_1_	1.11 (0.04)	[1.02, 1.2]	0.74 (0.01)	**<2^e-16^**
a_2_	0.67 (0.03)	[0.61, 0.73]	0.70 (0.02)	**<2^e-16^**
a_3_	0.57 (0.03)	[0.52, 0.63]	0.49 (0.02)	**<2^e-16^**
a_4_	0.47 (0.02)	[0.43, 0.51]	0.48 (0.02)	**<2^e-16^**
Cross-Lagged Paths				
b_12_	0.09 (0.02)	[0.05, 0.13]	0.07 (0.02)	**1.82^e-05^**
b_21_	0.01 (0.02)	[−0.02, 0.05]	0.01 (0.01)	4.72^e-01^
b_23_	0.04 (0.01)	[0.02, 0.07]	0.06 (0.02)	**1.72^e-03^**
b_32_	0.03 (0.02)	[0.00, 0.06]	0.03 (0.01)	4.44^e-02^

Note: Standardized β coefficients and unstandardized B coefficients are reported with standard errors (SE). 95% CIs are reported for unstandardized B coefficients. Bold *p* values are significant. All pathways are denoted by subscripts (see Figure 1).

**Table 1:** Demographic Information for the Sample at 7, 10, and 13 Years of Age

Demographics	Age 7 (n = 7,900)	Age 10 (n = 7,519)	Age 13 (n = 6,782)
Female %	49	50	50
Parental HE% (age 18+)	42	43	44
Owned/mortgaged home %	82	83	84
Ethnicity, white %	96	96	96
Full scale IQ, mean (SD) (Range: 45−151)^a^	105.30 (16.32)	105.24 (16.39)	105.70 (16.31)
Verbal IQ	108.14 (16.68)	108.08 (16.69)	108.51 (16.63)
Performance IQ	100.57 (16.94)	100.59 (17.03)	100.98 (16.93)

Note: IQ age 7 (n = 5,829), 10 (n = 5,761), and 13 (n = 5,307) years. HE = higher education; SD *=* standard deviation.

a Full range of scores at all ages.

**Table 2:** Parent-Reported Child Characteristics on Questionnaire Data

Questionnaires	Age 7 y (n = 7,900)	Age 10 y (n = 7,519)	Age 13 y (n = 6,782)
	Mean (SD) [CI]	Mean (SD) [CI]	Mean (SD) [CI]
SCDC	2.8 (3.66)	2.37 (3.58)	2.52 (3.60)
(Range: 0−24)^a^	[2.72, 2.88]	[2.29, 2.45]	[2.44, 2.61]
DAWBA-SF	0.88 (1.6)	0.98 (1.7)	1.26 (1.91)
(Range: 0−12)^a^	[0.85, 0.92]	[0.94, 1.02]	[1.21, 1.30]

Note: DAWBA-SF = Development and Wellbeing Assessment–Social Fears; SCDC = Social Communication Disorders Checklist; SD *=* standard deviation.

a Full range of scores at all ages.

**Table 3:** Correlation Coefficients Among All Latent Factors in the Saturated Model

	R [CI]					
	1	2	3	4	5	6
1. SA_7_	1					
2. SC Diff_7_	0.20∗∗∗ [0.17, 0.24]	1				
3. SA_10_	0.54∗∗∗ [0.51, 0.58]	0.21∗∗∗ [0.18, 0.25]	1			
4. SC Diff_10_	0.16∗∗∗ [0.12, 0.19]	0.74∗∗∗ [0.72, 0.77]	0.23∗∗∗ [0.19, 0.26]	1		
5. SA_13_	0.40∗∗∗ [0.36, 0.44]	0.17∗∗∗ [0.14, 0.21]	0.52∗∗∗ [0.49, 0.56]	0.20∗∗∗ [0.16, 0.23]	1	
6. SC Diff_13_	0.14∗∗∗ [0.11, 0.18]	0.61∗∗∗ [0.58, 0.65]	0.19∗∗∗ [0.16, 0.23]	0.71∗∗∗ [0.68, 0.74]	0.22∗∗∗ [0.19, 0.26]	1

Note: Subscript numbers show the age at assessment. SA = social anxiety symptoms; SC Diff = social and communicative difficulties.

∗∗∗ *p* < .001.

## Supplement 1

### Preliminary Analyses

In accordance with Avon Longitudinal Study of Parents and Children documentation for handling data, all variables were recoded, and total scores were created using the following prorating system. Individuals with more than 50% missing data on the Development and Wellbeing Assessment—Social Fears subscale (DAWBA-SF; >4 items) and Social Communication Disorders Checklist (SCDC; >7 items) at 7, 10, and 13 years of age were excluded from the specific time point and were not included in further analyses. Data checks were performed to assess outliers, and none were identified. Normality checks using skewness and kurtosis values revealed that several total score variables and items were not normally distributed; nonparametric tests were used as necessary. In all structural equation modeling analyses, a robust maximum likelihood (MLR) estimator was used to account for the data’s deviations from normality. Full information maximum likelihood (FIML) was used to account for incomplete data missing at random (MAR). The final analytic sample consisted of data from 9,491 children at 7, 10, and 13 years of age.

## Supplement 2

### Selective Attrition

Mann−Whitney *U* tests were carried out to investigate the selective attrition of social anxiety (SA) symptoms and social and communication (SC) difficulties. Group score differences on SA symptoms and SC difficulties were compared between those at age 7 years who had available and unavailable data at age 13 years. No significant difference in the SA symptoms at age 7 was found for those who had available (Median [Md] = 0, SD = 1.56) and unavailable (Md = 0, SD = 1.71) SA symptom data at age 13 (*W =* 6521400, *p* = .50, *d* = 0.02). A small but significant difference in SC difficulties at age 7 for those who had available (Md = 2, SD = 3.50) and unavailable (Md = 2, SD = 4.21) data at age 13 (*W* = 6032100, *p* = 6.88^e-04^, *d* = 0.13) was observed, with more SC difficulties present in those at age 7 who had unavailable data at age 13.
